# Fondaparinux: another potential treatment for heparin-induced thrombocytopenia type II?

**DOI:** 10.1186/s13054-016-1196-6

**Published:** 2016-01-27

**Authors:** Patrick M. Honore, Rita Jacobs, Inne Hendrickx, Elisabeth De Waele, Viola Van Gorp, Herbert D. Spapen

**Affiliations:** ICU Department, Universitair Ziekenhuis Brussel, Vrije Universiteit Brussel, 101 Laarbeeklaan, 1090 Jette Brussels, Belgium

Heparin-induced thrombocytopenia (HIT) type II is a highly morbid and potentially life-threatening condition with limited treatment options in older patients at high risk of bleeding who develop acute kidney injury (AKI). The recent study by Tardy-Poncet et al. [1] showing that argatroban may be a safe and valid therapeutic option in this patient population is therefore of utmost clinical importance. However, when discussing other alternative therapies for HIT type II, the authors did not mention recent experience with fondaparinux, a selective synthetic antithrombin-mediated inhibitor of coagulation factor Xa [2].

Fondaparinux has often been used off-label to treat HIT type II in patients without AKI. Maximal treatment efficacy was obtained in all patients at an approximately sixfold less bleeding risk than in subjects treated with direct thrombin inhibitors (DTIs), including argatroban [3]. As compared with DTIs, fondaparinux therapy is also more user-friendly (intermittent subcutaneous injections instead of continuous infusion) and less expensive. Fondaparinux is predominantly cleared renally. However, accumulation, and hence toxicity, is not expected to occur in patients undergoing renal replacement therapy (RRT). Indeed, because of its low molecular weight (1.7 kDa) and lack of binding to proteins other than antithrombin, fondaparinux should be eliminated easily by all currently used intermittent and continuous RRT techniques in critically ill patients [4]. Moreover, fondaparinux may form a perfect combination with regional citrate anticoagulation to reduce clinical but also dialysis circuit and filter thrombosis [5]. Of course, appropriate guidelines for dose finding, dose modification, and efficacy monitoring of fondaparinux during RRT must be elaborated.

## Abbreviations

AKI: Acute kidney injury
DTI: Direct thrombin inhibitor
HIT: Heparin-induced thrombocytopenia
RRT: Renal replacement therapy

## References

[1] Tardy-Poncet B, Nguyen P, Thiranos JC, Morange PE, Biron-Andréani C, Gruel Y (2015). Argatroban in the management of heparin-induced thrombocytopenia: a multicenter clinical trial. Crit Care.

[2] Brown P, Jay R, Fox A, Oliver M (2013). Chronic fondaparinux use in a hemodialysis patient with heparin-induced thrombocytopenia type II and extracorporeal circuit thrombosis—a case report and review of the literature. Hemodial Int.

[3] Efird LE, Kockler DR (2006). Fondaparinux for thromboembolic treatment and prophylaxis of heparin-induced thrombocytopenia. Ann Pharmacother.

[4] Sharathkumar AA, Crandall C, Lin JJ, Pipe S (2007). Treatment of thrombosis with fondaparinux (Arixtra) in a patient with end-stage renal disease receiving hemodialysis therapy. J Pediatr Hematol Oncol.

[5] Kalicki RM, Aregger F, Alberio L, Lammle B, Frey FJ, Uehlinger DE (2007). Use of the pentasaccharide fondaparinux as an anticoagulant during haemodialysis. Thromb Haemost.

[6] Cegarra-Sanmartin V, Gonzales-Rodriguez R, Paniagua-Iglesis P, Santamaria-Ortiz A, Cueva L, Galan-Serrano J, Moral-Garcia V (2014). Fondaparinux as a safe alternative for managing heparin-induced thrombocytopenia in postoperative cardiac surgery patients. J Cardiothorac Vasc Anesth.

[7] Pappalardo F, Scandroglio A, Maj G, Zangrillo A, D’Angelo A (2010). Treatment of heparin-induced thrombocytopenia after cardiac surgery: preliminary experience with fondaparinux. J Thorac Cardiovasc Surg.

[8] Warkentin TE, Pai M, Sheppard I, Schulman S, Spyropoulos C (2011). Fondaparinux treatment of acute heparin-induced thrombocytopenia confirmed by the serotonine-release assay: a 30-month, 16-patient case series. J Thromb Haemost.

