# Supplementary material for: Fondaparinux: another potential treatment for heparin-induced thrombocytopenia type II?
Source: Crit Care. 2016 Jan 27;20:14. doi: 10.1186/s13054-016-1196-6 (PMC4729072; doi:10.1186/s13054-016-1196-6)
Supplement: Related texts

# Reply: Authors’ response

**Authors:** Brigitte Tardy-Poncet, Bernard Tardy

We read with interest the letter by Honore et al. Even if our study [1] is the first prospective study, there are numerous retrospective studies that reported the efficacy of argatroban in the acute phase of HIT in ICU patients with renal failure and/or hemorrhagic risks. We have not quoted fondaparinux as a therapeutic option because the clinical experience of fondaparinux in HIT patients is still limited despite further previous studies [6–8], especially in ICU patients.

Questions remain regarding the efficacy, safety, and optimal doses of fondaparinux (Table 1). The very long half-life (18 h) of fondaparinux and its accumulation in the case of renal failure both represent major problems in critically ill patients, notably in those with hemorrhagic risk. Indeed, a major bleeding rate of 22 % (six cases among 27) is observed in HIT patients with a high hemorrhagic risk, such as those post cardiac surgery with renal failure (Table 1). Regarding argatroban, continuous infusion cannot be considered a handicap in ICU patients. Moreover, its price can be much reduced when the 250 mg/250 ml argatroban vial is fractionated (under a laminar fluid) in five vials containing 50 mg/50 ml each that will be used over 5 days.

**Table 1:** Heparin-induced thrombocytopenia patients with/without renal failure and treated with fondaparinux after cardiac surgery

Reference	N	Post cardiac surgery (n)	Renal failure (n)	HIT-associated thrombosis (n)	Major hemorrhage (n)	Duration (days)	Dose (mg/day)
[6]	15	15	14^a^	2	2	8–35	1.5–7.5
[7]	11	11	10^b^	3	3	4–17	2.5–7.5
[8]	16	11	3^c^	9	1	2–35	2.5–15

*HIT* Heparin-induced thrombocytopenia

^a^Eleven patients with creatinine clearance <30 ml/min

^b^Eight patients with creatinine clearance <30 ml/min

^c^One patient with creatinine clearance <30 ml/min

We do agree that fondaparinux could be used as an anticoagulant during hemodialysis. However, there are no convincing data allowing the use of fondaparinux to prevent or treat clinical thrombosis in the acute phase of HIT, especially in ICU patients.
